# A biomechanical comparison of Kirschner-wire fixation on fracture stability in Salter-Harris type I fractures of the proximal humeral physis in a porcine cadaveric model

**DOI:** 10.1186/s12917-017-1225-y

**Published:** 2017-10-25

**Authors:** Jiawen Ma, Tian Wang, Vedran Lovric, Kenneth A. Johnson, William R. Walsh

**Affiliations:** 1 Sydney School of Veterinary Science, Faculty of Science, University of Sydney, Sydney, NSW 2006 Australia; 2 Surgical and Orthopaedic Research Laboratories (SORL), Prince of Wales Hospital, University of New South Wales, Sydney, Australia

**Keywords:** Porcine physeal fracture, Salter Harris 1, Kirschner wire, Motion analysis tracking

## Abstract

**Background:**

The physis is the weakest component of immature long bones, and physeal fractures constitute about 30% of fractures in growing dogs. Fractures of the proximal humeral physis typically have a Salter Harris type I or II configuration. These fractures require accurate reduction and adequate stabilization to allow for any potential continued longitudinal bone growth, in conjunction with physeal fracture healing. Conventional internal fixation of these fractures involves insertion of two parallel Kirschner wires, although other methods described include tension band wiring, Rush pinning, and lag screws. However these recommendations are based on anecdotal evidence, and information about the biomechanical stability of physeal fracture repair is sparse. The unique anatomical structure of the epiphyseal-metaphyseal complex makes the gripping of the epiphysis for ex vivo biomechanical testing of physeal fracture repair very challenging. The objective of our study was to biomechanically assess the optimal number (three, two or one) of implanted Kirschner wires in a porcine Salter Harris I proximal humeral physeal fracture model, using motion analysis tracking of peri-fragmental retro-reflective markers while constructs were subjected to a constant axial compression and a sinusoidal torque of +/− 2 Nm at 0.5 Hz for 250 cycles.

**Results:**

There were significant differences between the three constructs (three, two or one Kirschner wire repair) for gross angular displacement (*p* < 0.001). The difference between three pins and two pins on toggle was not significant (*p* = 0.053), but both three-pin and two-pin fixation significantly reduced rotational toggle compared to one-pin fixation. Construct stiffness was not significantly different between any of the pin groups (*p* > 0.33).

**Conclusions:**

Motion analysis tracking using peri-fragmental markers in this porcine model of physeal fracture repair found that the stability at the fracture site of one-pin fixation was significantly less than two-pin and three-pin fixation. Whether there was increased stabilization of these fractures with three-pin fixation compared to two-pin fixation was not conclusive in this porcine model.

## Background

Physeal fractures occur in young animals prior to physeal closure and they constitute 30% of fractures occurring in growing dogs [1, 2]. Eight percent of all canine fractures involve the humerus [3]. A retrospective study of 130 humeral fracture cases in dogs and cats found that 4% of these fractures were proximal physeal fractures [4]. Proximal humeral fractures tend to occur due to vehicular trauma [5]. Closure of the proximal humeral physis occurs at 10–13 months of age and it is one of the last physes to close in the canine appendicular skeleton [6–8].

The proximal humeral epiphysis in the dog is formed through fusion of the cranial greater tubercle and caudal humeral head ossification centres, at an angle of approximately 102 degrees [8]. In fractures of the proximal humeral physis, there is usually concurrent separation of the humeral head and the greater tubercle from the humeral metaphysis [5, 9]. These fractures are typically Salter-Harris type I or II [9] and most require surgical intervention involving open reduction of the fracture followed by internal fixation [9].

The long-term prognosis for return to complete function of the physis is dependent on the degree of displacement, the accuracy of fracture reduction, the amount of interfragmentary motion, the age of the animal, and the physeal zones interrupted by the fracture [10]. Physeal fractures tend to occur at the hypertrophic zone due to the high cell to matrix ratio [1]. A good prognosis usually follows these fracture patterns as endochondral ossification may continue after the fracture is reduced [1]. If the fracture line crosses the proliferative zone (the epiphyseal-physeal border), then healing might be impaired by the formation of vertical septa and, subsequently, an epiphyseal bone bridge, which may result in premature closure of the physis [1]. The proximal humeral physis contributes 80% of the total longitudinal growth of the humerus [7]. As such, premature closure of the proximal humeral physis, especially before 6 months of age [7], may cause angular, torsional or axial length deformities which may result in substantial disparity in limb length.

The ideal fixation method of proximal humeral physeal fractures is not established. Currently, there is a lack of comparative insight into the biomechanical effectiveness of internal fixation methods for proximal humeral physeal fractures. Conventional internal repair of these fractures involve the insertion of two parallel Kirschner wires or small Steinmann pins directed distocaudally from the greater tubercle, through the physeal fracture line and into the caudal region of the proximal metaphysis [9]. A modification of this method has been described where a pin and tension band wiring combination is used [5]. Alternatively, double Rush pinning with craniomedially and craniolaterally placed pins through the greater tubercle has been stated to be the preferred method of repair [2]. It has also been suggested that the eccentric placement of the pins provides reduced stability at the humeral head and a proximo-caudally directed lag screw should be placed [5]. In contrast, it has been recommended that transphyseal tension band wires, screws and bone plates should be avoided as they cause physeal compression and may result in premature arrest of the physis with subsequent growth deformity [2]. However, these recommendations are all based on anecdotal evidence because the degree of stability with regards to internal fixation that will allow residual bone growth in proximal humeral growth plate fractures in the dog is unknown.

The purpose of this study was to create a reproducible Salter-Harris type 1 fracture model of the proximal humerus in cadaveric porcine bones and biomechanically test the effect of Kirschner wire fixation on this fracture model under peri-physiological torsional loading conditions. For this study we elected to use porcine bones harvested from cadaveric skeletally immature pigs what were all of similar size and age, because of the homogeneity of the available bone specimens, and their anatomical similarity to the humerus of dogs [11]. The specific aim was to compare the torsional stiffness and gross rotational displacement for physeal fractures fixed with three, two, and one transphyseal pins. Another aim was to compare the data from a servo-hydraulic material testing machine against the infrared motion analysis results for gross angular displacement. We hypothesised that a decrease in the number of Kirschner wire implants results in a decrease in torsional stiffness and increase in gross angular displacement. We further hypothesised that the motion analysis system yields more accurate absolute biomechanical data.

## Results

### Gross angular displacement

**Fig. 1:**
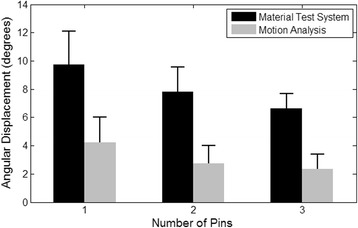
Comparison of mean ± SD angular displacement when specimens were fixed with a varying number of pins. Further comparison of mean angular displacement calculated from the materials testing system and motion analysis systems. The mean values for pin groups with different lower case letters are significantly different (*p* < 0.05)

No pins were visibly plastically deformed after testing and none of the specimens failed under the loading conditions. Motion analysis data showed a significant difference in torsional displacement between the three-pin group (2.34 ± 1.06°), two-pin group (2.75 ± 1.26°), and one-pin group (4.22 ± 1.79°) (Fig. 1 and Table 1). The one-pin group had 1.53 and 1.8 times greater gross angular displacement compared to the two-pin group and three-pin group, respectively. This was reflected by a significant difference in rotational displacement between the pin groups (*p* < 0.001). Subsequent pairwise comparison of the pin groups showed a statistically significant difference in torsional displacement of the three-pin and one-pin constructs (*p* < 0.001). The three-pin constructs had 1.18 times less rotational movement compared to the two-pin constructs (*p* = 0.012). There was also a difference in rotational displacement for fractures fixed with two pins and those fixed with one pin (*p* < 0.001). The rotational movement for cycles 60, 120, and 180 were 3.05 ± 1.58°, 3.12 ± 1.63° and 3.14 ± 1.64°, respectively. The cycle number had no significant effect on gross torsional movement (*p* = 0.315).

**Table 1:** Results for parameter values (mean +/− standard deviation) for all cycles (0–250) in relation to pin groups

Parameter	Collection Method	Number of Fixation Pins		
		Three	Two	One
Stiffness (N-m/^o^)	MTS^*^	1.18 ± 0.21^a^	1.10 ± 0.23^a^	1.16 ± 0.35^a^
Toggle (^o^)	MTS	4.25 ± 1.05^a^	5.08 ± 1.63^a^	6.41 ± 2.05^b^
Gross Angular	MTS	6.64 ± 1.03^a^	7.81 ± 1.73^a^	9.74 ± 2.36^b^
Displacement (^o^)	Motion Analysis	2.34 ± 1.06^e^	2.75 ± 1.26^f^	4.22 ± 1.79^g^

Within each row, values that have different superscript letters are significantly different (*p* < 0.05). Within the columns of gross angular displacement data, values that have different superscript letters are significantly different (*p* < 0.05)

*MTS = materials testing system

Data gathered from the materials testing system showed similar, but not identical, differences among the pin-groups as the motion analysis data. The angular displacement of fractures fixed with three pins (6.64 ± 1.03°) and two pins (7.81 ± 1.73°) were not significantly different. However, both the three pin and two pin groups were significantly different to the fractures fixed with one pin (9.74 ± 2.36°) (*p* < 0.001). The displacement data gathered from the material testing system were also of significantly greater magnitude than the calculated displacement values from the motion analysis system (*p* < 0.001) (Fig. 1, Table 1).

The cranial epiphyseal marker and, as such, the cranial surface of the epiphysis had a mean rotational displacement of 2.08 ± 1.42°. This was different from the mean movement tracked by the lateral marker (3.12 ± 1.38°), the caudal marker (3.84 ± 1.63°) and the medial marker (3.36 ± 1.51°) (*p* < 0.001). There was also a difference between caudal marker movement and the lateral and medial marker movements (*p* = 0.001 and *p* = 0.035, respectively). There was no significant difference in rotational motion between the lateral and medial aspects of the humerus (*p* = 0.244).

### Construct stiffness

**Fig. 2:**
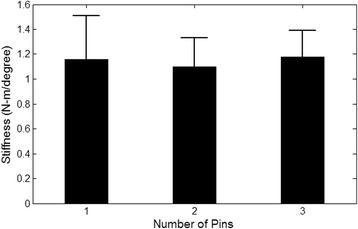
Comparison of torsional stiffness based on the number of pin implants. The differences between pin groups were not significant (*p* > 0.05)

Bones fixed with three pins and two pins had a mean stiffness of 1.18 ± 0.21 Nm/° and 1.10 ± 0.23 Nm/°, respectively. The mean stiffness of the bones fixed with one pin was 1.16 ± 0.35 Nm/° (Fig. 2 and Table 1). There was no difference in mechanical stiffness between the three pin and two pin groups (*p* = 0.33). Similarly, there was no difference in mechanical stiffness between the three pin and one pin groups (*p* = 0.66), nor was there any difference between the two pin and one pin groups (*p* = 0.49). When the bones were subjected to positive/external rotation, the mean mechanical stiffness calculated was 1.18 ± 0.23 Nm/°. Mechanical stiffness in negative/internal rotation was 1.11 ± 0.30 Nm/°, which was not significantly different to the stiffness associated with external rotation (*p* = 0.068). The load cycle did not play a significant role in torsional stiffness values (*p* = 0.15).

### Interfragmentary movement

**Fig. 3:**
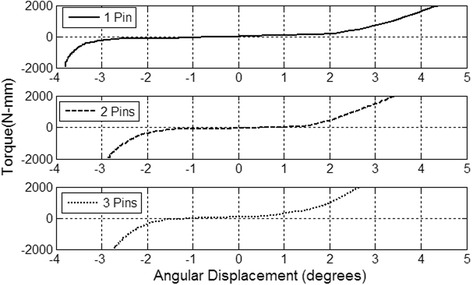
Comparison of the load-displacement curves for Specimen 9 fixed with one, two, and three pins

**Fig. 4:**
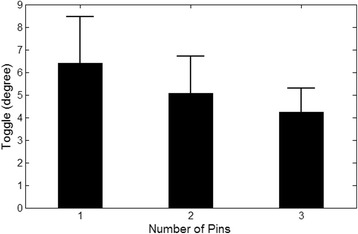
Comparison of toggle based on the number of pin implants. There was no significant difference in toggle between two-pin fixation and three-pin fixation (p > 0.05). There was a significant difference between one-pin fixation and the other groups (*p* < 0.05)

The mean interfragmentary movement associated with the ‘toggle’ effect was 5.23 ± 1.84°. Bones fixed with three pins had a mean toggle of 4.25 ± 1.05° compared to humeri fixed with two pins (5.08 ± 1.63°), and humeri stabilised with one pin (6.41 ± 2.05°) (Figs. 3 and 4). The toggle was significantly different between Salter-Harris type I proximal humeral fractures stabilised with three pins and one pin (*p* < 0.001). There was also a difference in the toggle associated with two pins and one pin (*p* = 0.004). Three-pin and two-pin fixation constructs had no significant variation in toggle (*p* = 0.053). The load cycle had no relationship to toggle when a comparison between the 60th cycle toggle and 120th cycle toggle was performed (*p* = 0.62). Similarly, a comparison between the 60th cycle and 180th cycle toggle generated a *P*-value of 0.62. The 120th and 180th cycles had no significant relationship regarding the toggle (*p* = 0.87). The mean toggle associated with a positive/externally directed torque (2.7 ± 1.26°) and negative/internally directed torque (−2.48 ± 1.36°) was not significantly different (*p* = 0.45).

## Discussion

A reproducible ex vivo physeal fracture model of the proximal humerus was created using cadaveric porcine bones. In this model, decreasing the number of Kirschner wire implants inserted into a Salter-Harris I physeal fracture of the proximal humerus significantly increased gross angular displacement of the epiphysis. However, decreasing the number of pin implants did not demonstrate the hypothesised decrease in rotational stiffness.

Gross rotational displacement was significantly different across all three of the pin groups when using the motion analysis method. There was a non-linear relationship between the number of pins and torsional motion, as depicted in Fig. 4. Based on motion analysis data, a decrease in stabilisation from three pins to two pins resulted in a 14.91% increase in gross torsional motion, with a further 34.83% increase when two pin fixation decreased to one pin fixation. In comparison, the materials testing system data showed a more linear relationship between pin number and angular displacement –decreasing the stabilisation from three pins to two pins resulted in a 14.98% increase in movement, and removal of an additional Kirschner wire resulted in a further 19.82% increase in movement. Displacement values retrieved from the materials testing system were 2.6 times greater than the displacement values calculated from motion analysis data. The difference between the data sets collected from both systems could be explained through the mild degree of slippage observed during our testing between the Wood’s metal mould and the proximal epiphysis which were held together by friction generated by the 20 Newtons of constant axial load.during testing. As motion analysis trackers were directly attached to the bones and, hence, directly tracked the amount of movement of the bone surface, the margin of error from the mould-bone interface could be assumed to have a lesser impact on motion analysis displacement data. As such, we have subsequently theorised that under torsional loading, biomechanical data generated from a motion analysis system provided data closer to the actual movement displayed by the specimens, compared to the material testing system.

There was no difference in rotational displacement between the selected load cycles. This suggested that there was no destabilisation over time, such as through frictional erosion of the bone at the interfragmentary interface of the physeal fracture gap or loosening of the Kirschner wires. This may be a consequence of the absence of true fatigue loading because 250 cycles of loading was equivalent to approximately 10 h of cage rest for a medium-sized dog [12]. This may not have been sufficient fatigue-loading to induce slippage of the fixation device and/or frictional erosion of the physis. The loading protocol for this study was selected to observe the degree of interfragmentary motion under peri-operative physiologic load conditions without fatigue loading.

The load-displacement curves showed the load parameters used in this model resulted in elastic deformation with no hysteresis or plastic deformation. There was no difference in torsional stiffness between any of the pin groups in this study. Other investigators have documented a rotational stiffness of 1.79 ± 0.87° under cyclic loading when a human three-part proximal humeral fracture was fixed with five 2.5 mm diameter Kirschner wires [13]. This value is 1.55 times greater than the mean torsional stiffness across all pin groups in our model. Interestingly, the total angular migration measured by Wheeler and colleagues (3.90 ± 3.10°) was 2.5 times less than the angular migration of the three-pin group in our study (materials testing system data comparison). The increase in angular migration in our study, despite a reduced and relatively static torsional stiffness between the pin groups, could be explained by the presence of the ‘toggle’ effect, or inherent interfragmentary instability. Hence, it can be supposed that the underlying impact of increasing the number of 3.0 mm Kirschner wires in this model was that it reduced the toggle, or degree of instability around the zero-load position, which subsequently affected the gross angular displacement without a significant effect on rotational stiffness.

Humeri stabilised with two pins had significantly less toggle movement compared to the same humeri stabilised with one pin. This difference coincided with the difference in gross angular displacement between these two groups. On comparison of the three-pin group against the two-pin group, interfragmentary motion was not different whereas there was a significant difference in gross angular displacement. This inconsistency between the measured biomechanical parameters for the comparison of three-pin fixation and two-pin fixation suggested that toggle was not the only factor contributing to gross angular displacement. The lack of difference between the three-pin and two-pin groups in the interfragmentary motion might also have been a type II statistical error dur to the small sample size used in our study, for example.

The authors of another study concluded that rigid fixation of a proximal physeal fracture in 6 to 7 week old rabbits resulted in fast ‘primary healing’ of the physeal separation [14]. There was initial angiogenesis of metaphyseal vessels across the separation gap and subsequent repair through continuation of normal endochondral ossification. Repair through secondary healing occurs if the metaphyseal vasculature is unable to cross the separation gap; in this situation fibrous tissue callus is deposited initially to increase fracture gap stability and this is followed by vascular ingrowth and restoration of normal endochondral ossification [14]. Factors such as a wide fracture gap and excessive interfragmentary movement will predispose to repair by secondary healing. The presence of the toggle effect in our load-deformation curves was suggestive of non-rigid fixation and may indicate secondary healing would be likely in an in vivo model. However, it is impossible to conclusively state a clinical route of physeal repair as repair is multifactorial with input of various factors ranging from fracture gap size to surrounding soft tissue contribution to fracture stability to the size of the surgical implant selected by the surgeon [15]. In our study, the decision to use Kirschner wires with a diameter of 3 mm was based on surgeon preference because definitive data on optimal pin diameter for physeal fracture stabilization are lacking. Experimental transphyseal drilling of holes across the rabbit distal femoral physis did not cause permanent growth disturbances provided that less than 7% of the cross-sectional area of the physis was destroyed by the drill holes [16]. We estimated that insertion of three 3-mm diameter Kirschner wires (combined cross-sectional area of 21mm^2^) would remove about 3% of the overall cross-sectional area of a physis with a diameter of 30 mm.

Increased toggle has been clinically related to increased pain perceived by the patient post-operatively [17]. We can state that from a purely biomechanical perspective, it was demonstrated that in our porcine model, the fixation of physeal separation of the proximal humerus with three Kirschner wires did not significantly reduce the interfragmentary movement compared to the conventional two-pin fixation method, as detected by the materials testing system method. However, it cannot be definitely concluded that a three Kirschner wire fixation method will provide increased fracture stability over two Kirschner wires for a Salter-Harris I separation of the proximal humerus in a clinical setting.

The degree of rotational displacement measured by motion analysis tracking differed, depending on the aspect of the bone. We found that there was an increase in the quantified rotation around the humeral head compared to the greater tubercle which reinforced the hypothesis that eccentric placement of the Kirschner wires results in asymmetrical stabilisation in fractures of the proximal humeral physis [5]. Movement in the humeral head was due to the absence of direct stabilisation in this region of the epiphysis because normograde (proximal to distal) pin placement was not feasible due to the presence of articular cartilage covering the humeral head and the gleno-humeral joint. However, retrograde pin placement, starting more distally on the cranial cortex of the proximal humeral metaphysis, with a proximo-caudal pin trajectory towards the humeral head, may be possible in achieving greater stability of the humeral head [9]. The biomechanical impact of this warrants further studies.

One limitation in this study was solely testing with a combined axial compression-torsion protocol. Other potential testing loads could have included shear forces and bending testing. It had been suggested that combined loading is more representative of the complicated loading patterns in vivo, and combined testing is more likely to challenge implant design [12]. Nevertheless, alternate force loading and proper fatigue testing of the construct should be incorporated for future tests.

The use of skeletally immature pig bones derived from abattoirs for creation of a physeal fracture model ensured that the bones selected for biomechanical testing were homogeneous in size and age. The freezing of bones for short term storage prior to testing does not have a significantly adverse effect on mechanical properties of bone [18]. Comparative interspecies studies of pigs and dogs of similar age found that trabecular bone core samples had similar ash concentration, bone mineral content and volumetric bone density [19]. However, fracture stress of trabecular bone was lowest for humans and pigs, intermediate for dogs and cows, and highest for sheep [19]. Also the age of pigs affects the mechanical properties of porcine bone, therefore the absolute results of our mechanical testing are not necessarily transferable to clinical patients of other species [20]. Furthermore, although the humerus of young pigs and dogs have similar proximal physeal morphology, it should be acknowledged that the columnar arrangement of proliferating chondrocytes in the physes of fast-growing domestic pigs is less distinctly organized, than those in the wild hog [11, 21]. This may render the physes of domestic pigs more susceptible to fracture, but we would not expect this to have influenced the results of our study.

Although a decrease in the number of Kirschner wire insertions should have resulted in a decreased stiffness [13], it was not demonstrated in this study. This may be due to data gathered from physiologic fatigue loading conditions as opposed to loading to failure. However, even in the sub-failure cyclic loading environment, calculation of the slope from a terminal region should provide internally comparable stiffness values, as evidenced from other studies [12, 17]. The entire process of calculating stiffness used in our study was standardised according to the method used by others [12]. Although the correlation (R^2^) value for the linear regression fitted to the terminal linear inclines of the curve were set at ≥0.995, the length of the linear terminal slope was relatively short (range of seven – twenty-two points). Some runs may not have yielded a sufficient number of linear points to accurately approximate a slope and, hence, precise torsional stiffness measurements.

## Conclusions

No other reports of biomechanical studies are present in the current literature which analyse the effect of increasing the number of Kirschner wire implants for the fixation of a reproducible proximal humeral physeal fracture model. This repeatable porcine fracture model offered the inherent stability of the physeal topography alongside the stability offered by the fixation implant. This model demonstrated that decreasing the number of Kirschner wires used to stabilise a physeal separation of the humeral epiphysis significantly increased the amount of gross angular displacement measured by the motion tracking analysis method, under axial compression-torque combination loading. It was further concluded that interfragmentary motion was only reduced to a degree and there was no further reduction in toggle when an extra pin implant was included on top of the conventional two wire fixation. The lack of an increase in torsional stiffness along with a reduction in interfragmentary motion suggested that two Kirschner wires provides similar stability to three Kirschner wires without the additional implant material in Salter-Harris I proximal humeral fractures. Further clinical studies are necessary to assess the clinical significance of using a three-wire fixation method compared to two wires in patients with Salter-Harris type I proximal humeral fractures.

## Methods

### Specimen preparation

Twelve cadaveric porcine right humeri were randomly sourced from pigs slaughtered at accredited abattoirs and thereafter butchered and deboned for retail sale. The donor animals were 6-8 months of age and the size and shape of the humeri were similar. Soft tissue was stripped from the bones using size 22 scalpel blades and a periosteal elevator. Cranio-caudal and lateral radiographs were obtained of each specimen with a specimen radiography system.^1^ Individual bones were wrapped in two layers of absorbent paper^2^ soaked in phosphate buffered saline, stored in a resealable plastic bag and labelled, and stored in a freezer at −20 °C.

### Experimental fracture creation

**Fig. 5:**
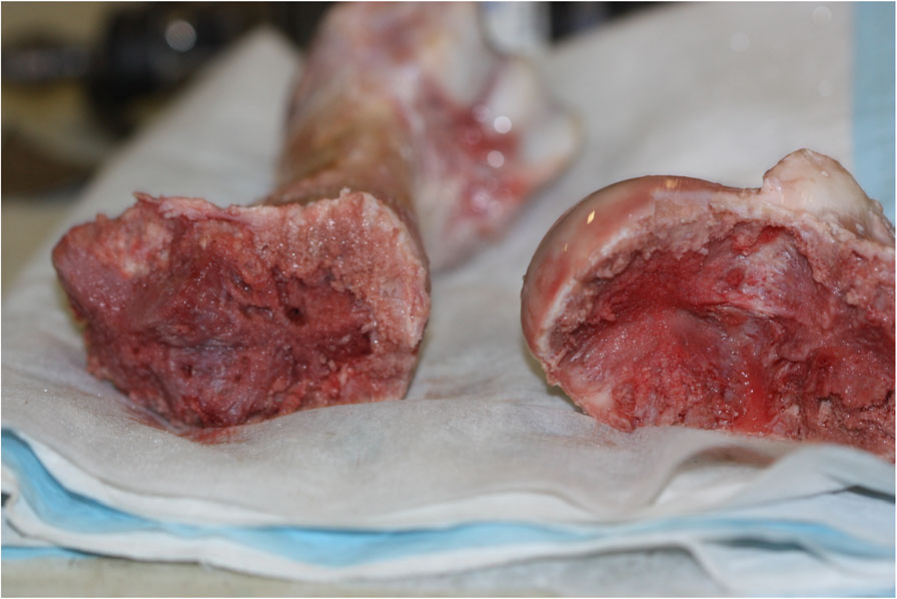
Creation of a reproducible Salter-Harris I fracture of the proximal humeral physis resulting from separation of the conjoined epiphyses of the humeral head (H) and the greater tubercle (T)

A reproducible Salter-Harris I fracture of the proximal physis was created by initial burring along the perichondrial margins of the exposed physis with a rotary tool with a burr attachment.^3^ A small slotted screwdriver and hammer were subsequently used to produce gradual circumferential separation of the physis. When enough instability was achieved, the combined epiphyses of the humeral head and greater tubercle were lifted off as a single unit and detached completely. This resulted in a reproducible Salter-Harris type I fracture (Fig. 5).

### Fracture stabilisation

**Fig. 6:**
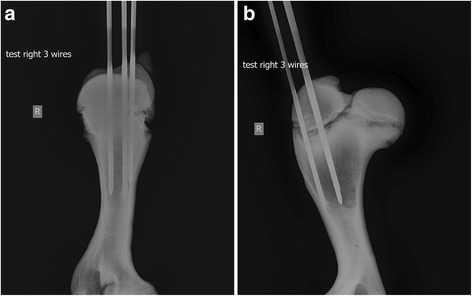
Orthogonal cranio-caudal (**a**) and latero-medial (**b**) projection radiographs of a prototype pre-test bone. Note the cortical engagement that was not avoided in this prototype test

Trocar pointed Kirschner wires of 3.0 mm diameter^4^ were selected for fixation, based on surgeon experience. These were inserted using a variable speed cordless power drill^5^ to a pre-marked depth of 80 mm. A standard 80 mm depth was ascertained after trial stabilisation in test samples to minimise the chance of trans-cortex engagement. These were manually measured and centred in the greater tubercle in an equilateral format – one cranial pin, one caudolateral pin and one caudomedial pin. The aim was to insert the pins parallel to each other and perpendicular to the physis without trans-cortex engagement. Medio-lateral and cranio-caudal radiographs were obtained after each pin placement to improve reproducibility by avoiding distal cortical engagement (Fig. 6).

### Construct assembly

**Fig. 7:**
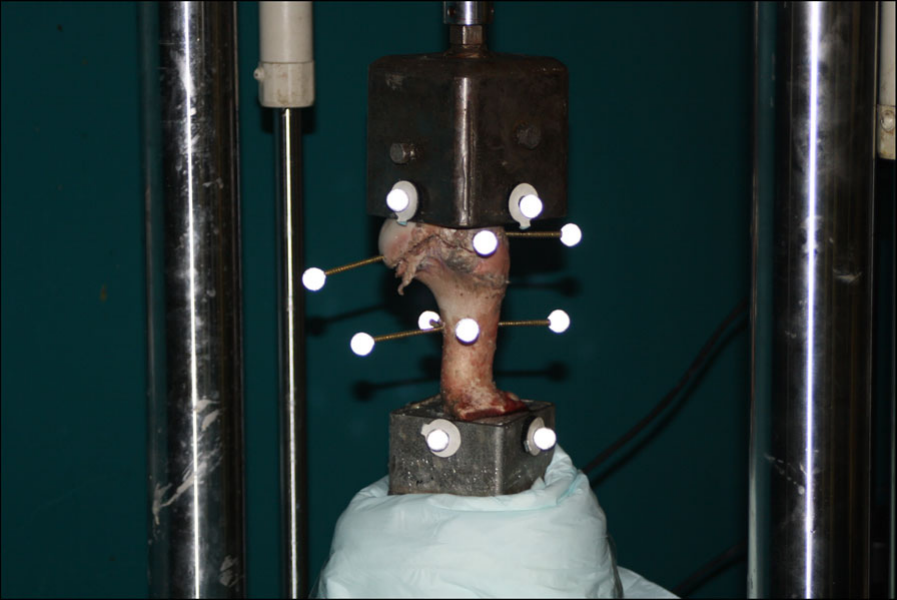
Specimen 6 loaded into the servo-hydraulic material tester with infrared markers attached

Each bone specimen was placed into vertically aligned metal pots and the distal end of the humerus was embedded into each pot with liquid Wood’s metal at >80 °C, which then hardened upon cooling to room temperature. Afterwards, the pin ends were wrapped in multiple layers of aluminium foil prior to insertion of the combined humeral head and greater tubercle epiphyses into the proximal pot containing liquid Wood’s metal. After solidification of the mould, the foil layers were discarded, effectively creating space between the Wood’s metal mould and the exposed pin ends to avoid physical interference of the mould with results. This precaution was taken to remove any potential stability offered by the mould on the fixation construct. Due to a shallow proximal epiphysis-mould interface, a 60 mm 8 gauge wood screw was inserted into the humeral head for larger surface area engagement within the mould. A proximal mould created for specimen 6 was re-used for all test specimens, as there was minimal anatomical variation between specimens, of the humeral head and greater tubercle. A 2.2 mm drill bit was used to pre-drill peri-fragmental holes in the cranial, caudal, lateral and medial aspects of both the proximal epiphysis and the proximal diaphysis. Retro-reflective markers were attached via 60 mm 8 gauge flathead wood screws to these sites – four on the epiphysis and four on the diaphysis. The markers were positioned cranially, caudally, medially, and laterally as accurately as possible although no method of standardisation was used between test specimens. Reference markers were also placed on each aspect of the two moulding pots (Fig. 7).

### Mechanical testing

Each specimen was cyclically loaded in external and internal rotation by subjecting them to a torsional loading of ±2 Nm for 250 cycles at 0.5 Hz in a servo-hydraulic material testing machine.^6^ Due to the absence of physiological loading data in the literature for canine humeri, torque was selected based on the range of torque values used for cyclic loading studies in proximal humerus fractures in human literature, which has been estimated to be peri-physiological [13, 17, 22, 23]. Furthermore, a constant axial compression of 20 Newtons was implemented as a means of combined loading to be more representative of loading in vivo [12] by engaging the inherent stability offered by the irregular topography of the proximal physis in rotational testing. Each bone was initially fixed with three pins and after each run of 250 cycles, the actuator was elevated and a Kirschner wire was removed. As such, subsequent tests were repeated on the same bones with two pins (cranial pin removed), and eventually one pin (cranial and lateral pins removed).

### Data collection and analysis

Three-dimensional co-ordinate data were captured by six surrounding motion analysis infrared cameras^7^ and exported to motion capture software.^8^ A combination of the Euclidean formula with the Law of Cosines was used to calculate the rotational displacement (in degrees) of each epiphyseal marker. Rotational displacement for a full cycle for each epiphyseal marker at 60 cycles, 120 cycles, and 180 cycles was selected to assess the potential effect of frictional erosion of the physis and implant slippage on biomechanical parameters. A linear mixed effects model was created through stepwise removal of negligible random effects – the criterion used for determining omissible random effect factors was based on the statistical results from a Chi-square test comparison of the models. The number of pins was a fixed effect (due to standardisation of placement) whereas ‘bone’ and ‘marker’ were deemed to be factors exhibiting random effects. Refinement of the initial model resulted in a final mixed effects model:

$$ {y}_{ijkl}\sim {\beta}_0+{\beta}_1{X}_i+{\beta}_2{X}_j+{\gamma}_1{Z}_k+{\gamma}_2{Z}_l+{\varepsilon}_{ijkl} $$

where *β*_1_ and *β*_2_ are fixed effects, *γ*_1_ and *γ*_2_ are random effect with means of 0 and variances ${\sigma}_1^2$ and ${\sigma}_1^2$, respectively, *X*_*i*_ represents the factor for the number of pins, *X*_*j*_ represents the factor for the load cycle, and *Z*_*k*_ represents the factor for the specimen number and *Z*_*l*_, the epiphyseal marker factor.

The materials testing system data retrieved overall angular movement every millisecond. For comparison, the MTS values for maximum and minimum torsional displacement values were found for the 60th, 120th and 180th cycles. The maxima and minima rotational displacement were subsequently summed for each of these cycles to calculate the full cycle rotational displacement. A linear mixed effects model was also generated for this dataset to assess the significance of the number of pins and time on torsional displacement:

$$ {y}_{ijk}\sim {\beta}_0+{\beta}_1{X}_i+{\beta}_2{X}_j+{\gamma}_1{Z}_k+{\varepsilon}_{ijk} $$

where *β*_1_ and *β*_2_ are fixed effects, *γ*_1_ is a random effect with a mean of 0 and variance, ${\sigma}_1^2$, *X*_*i*_ represents the factor for the number of pins, *X*_*j*_ represents the factor for the load cycle and *Z*_*k*_ represents the factor for the specimen number.

For each of the aforementioned cycles, a bi-directional load-displacement curve (both external and internal rotation) was graphed using the materials testing system data (Fig. 3). The maximum and minimum torque points were found and the previous ten data points from both these points were picked up. Then iterative correlation tests were performed with an additional adjacent data point in the terminal slopes until an R^2^ value ≥0.995 was achieved [12, 24]. This protocol was established to standardise the selection of the dataset representing the terminal portion of the load-deformation curve from which external and internal rotational stiffness would be estimated. A regression line was established for each set of points and the regression coefficient was found. This coefficient represented the gradient of the linear regression of the terminal portions of the load-displacement curves, which was an estimation of the torsional stiffness (Nm/degree) of the construct in external and internal rotation.

The load-displacement curves were sigmoidal and showed considerable interfragmentary motion around the zero-load position for all torsional cycles (Fig. 3). This has been described by others as ‘toggle’, which refers to zero mechanical stiffness (slope of zero in the load-displacement curve) or gross allowable interfragmentary motion [17]. Calculation of the positive, negative, and total toggle displacement was performed by extending the linear model for internal and external rotation to the x-axis and calculating the difference between the x-intercepts [12].

Mixed effects linear regression modelling for both the materials testing system data and motion analysis data were used to compare the rotational displacement of each bone for each pin group with statistical software,^9^ loaded with the ‘lme4’ mixed effects model package. This statistical method was chosen to remove bias imposed from random effects. If statistical significance was observed for any factor in the model, a pairwise t-test comparison was selectively performed to observe the statistical significance between each of the levels. A two-sample t-test was performed on the materials testing system angular displacement data and motion analysis angular displacement data for a comparison of the results obtained by the two systems. A *P*-value ≤0.05 was deemed significant.

## Footnotes

1 Faxitron Bioptics, LLC

2 RediwipeTM, Cello Paper Pty Ltd., Sydney, Australia

3 Dremel Multipro 395TM

4 Synthes, Monument, Colorado, USA

5 Makita,

6 MTS, MTS Systems Corporation, Eden Prairie, MN, USA

7 Osprey Digital Realtime System, MotionAnalysis

8 Cortex, MotionAnalysis

9 R Project for Statistical Computing
